# Study of mental health of medical staff in a specialized hospital for COVID-19 in Novi Sad

**DOI:** 10.1192/j.eurpsy.2022.1384

**Published:** 2022-09-01

**Authors:** D. Kuljancic

**Affiliations:** University of Novi Sad Medical faculty, Psichiatry, Novi Sad, Serbia

**Keywords:** COVID19 pandemic, mental health, medical staff, affective symptoms

## Abstract

**Introduction:**

The highly infectious novel coronavirus disease (COVID-19) emerged in Wuhan, China in late 2019 and soon became a global pandemic. COVID-19 is escalating medical staff psychological stress and creating an increasingly heavy professional burden. Fear of transmitting the virus to family, community perception of frontline workers as potential disease carriers, extreme workloads and moral dilemmas add additional stressors. In Novi Sad Clinical Centre of Vojvodina (CCV) for the past 2 years there has been a continuous struggle against the COVID-19 crisis. Both senior specialist doctors and newly hired young doctors, some without work experience, were hired immediately after completing their studies.

**Objectives:**

To investigate the mental health of clinical first-line medical staff in COVID-19 pandemic.

**Methods:**

This is a cross-sectional study involving CCV staff who worked in the first line of patient treatment during the COVID-19 pandemic. They were given a self-administered questionnaire which included information on demographic and socio-economic characteristics and the validated Depression, Anxiety, and Stress Scales (DASS-21) and the Impact of Events Scale–Revised (IES-R) instrument. A total of 190 medical workers were involved.

**Results:**

Sixty-two (32,6%) participants screened positive for anxiety, 38 (20%) for depression, 68 (35,8%) for stress, and 22 (11,5%) for clinical concern of PTSD. The most endangered are young nurses and doctors with less than 6 months of previous work experience.

**Conclusions:**

In conclusion, our results suggest frontline medical staff involved in treatment of COVID-19 patients should be closely monitored as a high-risk group for depression and anxiety, and given proper training before deployment.

**Disclosure:**

No significant relationships.

